# Critical Solutal Marangoni Number Correlation for Short-Scale Convective Instabilities in Drying Poly(vinyl acetate)-Methanol Thin Films

**DOI:** 10.3390/polym13172955

**Published:** 2021-08-31

**Authors:** Max Tönsmann, Philip Scharfer, Wilhelm Schabel

**Affiliations:** Karlsruhe Institute of Technology (KIT), Thin Film Technology (TFT), Kaiserstraße 12, 76131 Karlsruhe, Germany; philip.scharfer@kit.edu (P.S.); wilhelm.schabel@kit.edu (W.S.)

**Keywords:** polymer film drying, Marangoni number, convective instability, film drying simulation, self-assembly

## Abstract

A new empiric correlation for the critical solutal Marangoni number as function of the Péclet and Schmidt numbers is proposed. It is based on previously published experimental flow field data in drying poly(vinyl acetate)-methanol films with an initial thickness in the range of 20–100 μm and an initial solvent load of 1 to $2{\;g}_{\mathrm{MeOH}}/g_{\mathrm{PVAc}}$, as well as newly derived concentration profile measurements and 1D drying simulations. The analysis accounts for realistic transient material properties and describes the occurrence of short-scale convective Marangoni (in)stabilities during the entire drying process with an accuracy of 9%. In addition, the proposed correlation qualitatively follows trends known from theory. As convective Marangoni instabilities in drying polymer films may induce surface deformations, which persist in the dry film, the correlation may facilitate future process design for either thin films with uniform thickness or deliberate self-assembly.

## 1. Introduction

Thin functional polymer films are used in many products, such as displays, organic light emitting diodes, biosensors, battery separators, or membranes for fuel cells. In order to ensure product functionality and high efficiencies, film thickness variations should not exceed 1% in many applications [1,2]. Since polymer films are commonly produced from solvent solution via coating and subsequent film drying, convective instabilities in the drying liquid film have the potential to induce free-surface deformations which persist in the dry polymer films [3]. Therefore, a fundamental knowledge regarding the impact of drying conditions on convective instabilities is desirable.

### 1.1. One-Dimensional Polymer Film Drying

The drying and shrinkage of polymer-solution films is typically regarded as a one-dimensional (1D) process perpendicular to the film plane, accounting for 1D solvent diffusion in the film, the phase equilibrium at the free surface and 1D solvent transport in the gas phase [4,5,6,7,8,9,10]. The phase equilibrium and the mutual mass diffusion coefficient of polymer solutions usually exhibit a low concentration dependency for diluted solutions with values close to the properties of the pure solvent, whereas low solvent concentrations lead to a strong decrease of said properties [6,7,11]. Polymer film drying of diluted solutions, therefore, results in an initial constant evaporation rate (constant rate period (CRP)) because the solvent transport is limited by the mass transport in the gas phase [4,12,13,14,15]. At lower solvent concentrations, the evaporation becomes limited by the solvent diffusion in the film and the evaporation rate decreases significantly [6,7,11]. Furthermore, the pure polymer may exhibit glass transition. An added solvent acts as a plasticizer, significantly decreasing the glass transition temperature [13,16,17]. When the glass transition temperature of the pure polymer is above the drying temperature of the film, the film may undergo a transition from rubbery to glassy state during drying, starting at the solvent depleted free surface [7,10,18,19]. This may lead to a glassy surface layer, inhibiting further surface tension gradient-induced convective Marangoni instabilities (see next section) due to the strong viscosity increase associated with glass transition [16,20,21,22].

In our own group, significant effort has been made in the past, regarding the experimental and numerical investigation of 1D polymer-solution film drying: A measurement technique based on Raman spectroscopy was developed, providing transient vertical concentration profiles in films under various drying conditions (Inverse Micro Raman Spectroscopy (IMRS)) [4,23,24]. The concentration- and temperature-dependent phase equilibrium of various binary polymer-solvent solutions, as well as the mutual mass diffusion coefficient, were measured using a sorption balance [12,25] and Raman drying experiments [11,24], respectively. Other authors used similar experimental methods or determined the diffusion coefficient from the free-volume theory [26].

Based on these material properties, a one-dimensional isothermal simulation model for polymer film drying was developed [27,28] and extended to non-isothermal drying conditions [29]. The transient vertical concentration profiles of several different polymer solution films could be successfully simulated with this model, assuming Fickian diffusion and a concentration-dependent diffusion coefficient [4,5,11,19]. Other authors additionally accounted for a visco-elastic contribution to diffusion, in order to model sigmoidal-shaped solvent concentration profiles [9,10,18].

### 1.2. Thermally Induced Marangoni Convection

Convective instabilities predominant in thin liquid films are due to surface-tension gradient-induced Marangoni convection. Bénard was the first to report regular patterns of vertical convection cells in non-evaporating pure liquid films heated from below, with a lateral length scale similar to the film height [30,31]. Pearson was the first to suggest surface-tension gradients as driving force of such instabilities [32]. He performed a theoretic linear stability analysis, assuming a non-volatile pure liquid film with non-deformable free surface heated from below. His analysis resulted in a dimensionless number, later to be named Marangoni number $Ma_{T}$, as a measure to assess whether films are convectively stable or instable. A lower limit for $Ma_{T}\geq 80$ was found, below which no cellular Marangoni instability may occur for a film with constant temperature at the bottom. This stability threshold was named critical Marangoni number $Ma_{T,crit}$. It has to be noted that $Ma_{T,crit}$ is not a constant value, but it depends on boundary conditions. According to Pearson’s analysis, it increases with increasing upper Biot number and the lower limit reduces to $Ma_{T}\geq 48$ when considering a constant heat-flux boundary condition at the bottom of the film. Pearson also suggested that buoyancy-driven convective instabilities are only relevant for a film thickness above 1 mm, which still appears to be common consensus in the present. By now, it is established to refer to the dimensionless Bond number Bo, in order to assess whether cellular convective instabilities are caused by buoyancy driven Rayleigh-Bénard convection (Bo≫1) or by surface-tension gradient-induced Bénard-Marangoni convection (Bo≪1) [33,34,35].

Scriven and Sternling extended Pearson’s model allowing for free-surface deformation of the film [36]. They found an additional mode of instability when the lateral length scale of perturbations significantly exceeds the film thickness, having a lower $Ma_{T,crit}$ limit. These modes of instability are today referred to as short-scale and long-scale Marangoni instability, respectively [37,38]. While surface deformability is no necessary condition for short-scale Marangoni instability, it is intrinsic for long-scale instabilities and may lead to partial dewetting [37,39]. Both instability modes may interact [38].

Pure liquid films subject to evaporation may also exhibit Marangoni instability even when cooler at the bottom, which should be stable according to Pearson’s and many following theoretical analyses. Zhang et al. reported experimental findings from evaporating thin films [40,41]. They conclude that evaporative cooling of the film surface alone cannot account for the observed instability. They further suggest that naturally occurring lateral perturbations in evaporation rate might have a direct impact on convective instabilities in evaporating pure liquid films. For more information regarding thermally-induced Marangoni convection, the readers are referred to several comprehensive reviews on the matter in References [35,39,42,43].

### 1.3. Marangoni Convection in Drying Polymer Films

An additional level of complexity arises when considering drying of polymer solutions: Primarily, the surface tension changes during the drying process, due to its dependency on temperature, as well as concentration [44]. In addition, the viscosity strongly increases during drying with decreasing solvent content dampening any flow, and the mass diffusion coefficient strongly decreases, especially for low solvent concentrations [4]. The latter is important, since solvent diffusion may mitigate concentration gradients and, therefore, solutally-induced Marangoni convection. Several authors have theoretically investigated solutal Marangoni convection in various configurations, but made simplifying assumptions, such as constant viscosity [45,46,47], constant diffusion coefficient [45,46,47,48], or a surface tension linear with concentration (all of the aforementioned references and References [49,50]). In addition, the effect of inter- and intra-molecular interaction on the (in)stability in ultrathin films with a thickness in the nanometer range (≤100 nm) was reported [35,51,52,53,54,55,56].

Existing experimental work regarding convective instabilities in drying polymeric films appears to be governed primarily by deliberate patterning, utilizing Marangoni convection as a means for self-assembly [57,58,59,60]. The transition between different modes of instability and lateral size and form of convection cells was also investigated [58,59,61,62,63,64,65,66,67]. When thermal and solutal gradients arise simultaneously, several authors conclude that the solutal effect is dominant [48,49,68]. Experimental data on the stability threshold of convective instabilities are, however, scarce. This has been acknowledged by several authors in the past [67,69,70]. Recently, Wang et al. explicitly emphasized the need for new quantitative experiments regarding convective instabilities in drying polymer films in a comprehensive review on multiphase Marangoni convection [70].

Bassou et al. observed short-scale convection cells in drying polystyrene-toluene films with an initial thickness in the range of 55 μm–1.5 mm, initial polymer concentrations in the range of 5–20 vol%, and an initial viscosity of 0.01–0.46 Pa⋅s [58]. They calculated the thermal and solutal Marangoni numbers at the onset of drying and found that the thermal Marangoni numbers were significantly below available critical thresholds for films with an initial thickness $h_{0}\leq 1\;\mathrm{mm}$ and an initial polymer concentration ≥15 vol%, whereas the solutal Marangoni numbers were significantly larger. This lead the authors to the conclusion that solutally-induced short-scale instabilities dominate over thermally-induced instabilities for thin films and large initial polymer concentrations.

Doumenc and coworkers published a series of findings regarding short-scale convective instabilities during polymer film drying: They reported experimental investigation of convective instabilities in drying polyisobutylene-toluene films with initial wet-film thickness in the range of 0.3 to 14.3 mm, an initial polymer mass fraction of 0–15 wt%, and an initial viscosity of 0.55–2100 mPa s [62]. For an initial film thickness $h_{0}\leq 4\;\mathrm{mm}$, they observed Bénard-like convection cells and for thicker films they found roll cells, persisting even after a solvent-depleted viscous surface layer emerged during drying. The authors attributed these modes of instability to Marangoni effects and buoyancy driven Rayleigh convection, respectively. In a follow-up work, Doumenc et al. derived a theoretic stability analysis accounting for thermally-induced Marangoni and buoyancy driven convective instabilities, including a realistic viscosity increase during polymer film drying [71]. The results were in reasonable agreement with their experimental findings at the onset of drying.

Doumenc et al. also investigated solutally-induced short-scale Marangoni instabilities numerically, accounting for a realistic viscosity increase based on experimental values from polyisobutylene-toluene solutions [72]. The following simplifying assumptions were made: The concentration dependency of the mutual diffusion coefficient was neglected, using a constant value of $D=10^{-10}\;m^{2}/s$, and the concentration dependency of the surface tension was assumed to be linear. In addition, they focus on diluted solutions and report no experimental validation. Nevertheless, their theoretical analysis revealed two interesting relations: First, the critical solutal Marangoni number $Ma_{s,crit}$ increases with increasing Péclet number $Pe_{int}=v_{int}h/D$, where $v_{int}$, h and D denote the velocity of the free film surface, the film thickness and the mutual mass diffusion coefficient, respectively. Second, $Ma_{s,crit}$ initially strongly decreases with increasing Schmidt number Sc, asymptotically approaching a constant value for very large Schmidt numbers. The reported numerically derived critical solutal Marangoni numbers $Ma_{s,crit}$ range in the order of 10^2^–10^5^.

In our group, the surface deformation of poly (vinyl acetate)-methanol films subject to deliberate laterally inhomogeneous drying conditions was investigated. A qualitative comparison with 1D non-isothermal drying simulations revealed the dominance of solutal effects over thermal effects for lateral long-scale convection [73]. In order to assess the driving forces of Marangoni instabilities, the concentration and temperature-dependent surface tension of several polymer solutions was experimentally investigated over the whole concentration range. It was found that all five binary polymer-solvent solutions under investigation show a non-linear concentration-dependent surface tension [44].

In addition, we have established a microscopic measurement technique, based on particle tracking velocimetry and designed for quantitative measurements of the transient three-dimensional flow-field in drying thin films (three-dimensional micro particle tracking velocimetry, 3D-µPTV) [74,75]. The line-of-sight tracer particle position can be reconstructed by correlating the diffraction ring pattern of particles not in the lateral focus plane to different vertical positions, which was originally proposed by Speidel et al. as “off-focus imaging” [76]. This approach allows for 3D flow field measurements with a single camera, but the vertical extent of the observation volume of a single camera is limited due to deteriorating signal-to-nose ratio with increasing diffraction ring size. Therefore, 3D-µPTV combines “off-focus imaging” with multifocal microscopy, utilizing several cameras with different vertical focus plane positions [74,75].

**Figure 1:**
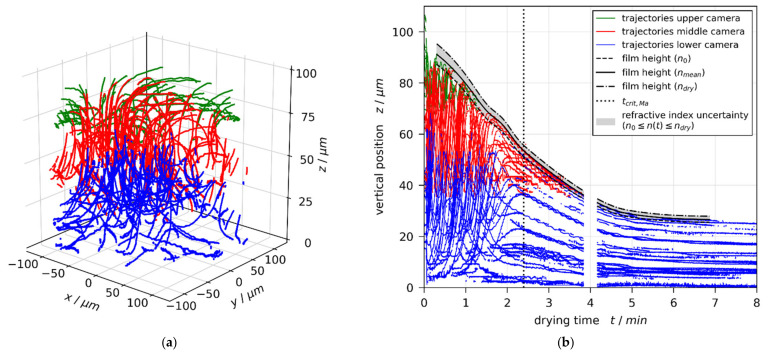
Flow field in a poly (vinyl acetate)-methanol film ($X_{0}=1.5\;g_{\mathrm{MeOH}}/g_{\mathrm{PVAc}}$ and coating gap $h_{gap}=200\;\mu m$), drying at $T_{substrate}=20\;{}^{\circ}C$ and otherwise ambient conditions, measured with µPTV and evaluated with mean refractive index. The flow field clearly shows convective instabilities. (**a**) 3D tracer-particle trajectories. (**b**) Transient vertical tracer particle positions. The convective instability stops during drying at $t_{crit,Ma}=144\;s$. Since the reconstruction of vertical particle positions from observed diffraction rings depends on the refractive index of the drying film, the extracted drying curve (black curves) is additionally given for an evaluation with the refractive index of the coating solution $n_{0}$ and the dry polymer $n_{dry}$, respectively. Reprint from [1], License: CC-BY 4.0.

Based on this measurement technique, we recently reported short-scale Bénard–Marangoni convection cells in drying poly (vinyl acetate)-methanol (PVAc-MeOH) films with an initial thickness of approximately 20–100 μm and an initial solvent load of 1–2${\;g}_{\mathrm{MeOH}}/g_{\mathrm{PVAc}}$ (50–67 wt%), drying at a substrate temperature of 20 °C and otherwise ambient conditions [1]. It was found that all investigated films with an initial solvent load of $1{\;g}_{\mathrm{MeOH}}/g_{\mathrm{PVAc}}$ were convectively stable and the flow field solely exhibited vertical film shrinkage during the entire drying time. An initial solvent load of 1.5 and $2{\;g}_{\mathrm{MeOH}}/g_{\mathrm{PVAc}}$, however, resulted in short-scale convection cells, emerging at the start of drying. The convective instability stopped at different critical threshold times $t_{crit,Ma}$ during drying, followed by continued 1D film shrinkage. Figure 1 shows exemplarily the 3D tracer particle trajectories (Figure 1a) and the transient vertical tracer particle positions (Figure 1b) in a drying film, initially exhibiting short-scale convective instabilities.

From an analysis of the velocity distribution and from a coarse assessment of the upper limits of the Bond number ($Bo_{max}=5\cdot 10^{-3}\ll 1$), as well as the thermal and solutal Marangoni numbers ($Ma_{T}<6$, $Ma_{s}<11,429$), we deduced that the found convective instability were solutally driven Bénard-Marangoni convection [1].

The aim of this work is the quantitative stability assessment of these recently published 3D-µPTV derived flow field results in the form of critical Marangoni numbers, taking into account initial conditions, as well as the critical threshold time $t_{crit,Ma}$ at which convective instabilities stopped during drying. We account for realistic material properties, especially regarding surface tension, viscosity and mass diffusion coefficient. It will be shown that vertical concentration profiles, as well as drying curves (transient film height), from one-dimensional drying simulations, are in good agreement with experiments, independent of initial (in)stability. Combining the information on convective (in)stability of the already published experimental flow-field results [1] with newly derived transient thermal and solutal Marangoni numbers, calculated from simulations, gives access to realistic critical Marangoni numbers.

## 2. Materials and Methods

### 2.1. Materials, Solution Preparation, Coating, and Drying

**Figure 2:**
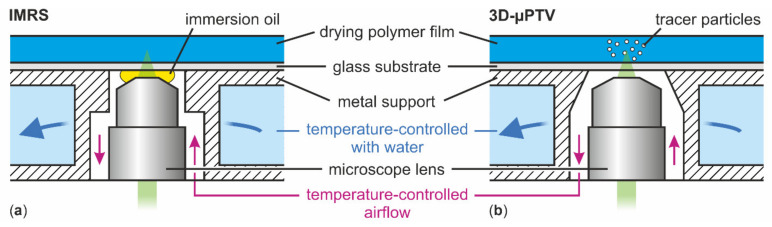
Schematic drawing of the substrate temperature control during IMRS (**a**) and 3D-µPTV (**b**) drying experiments. The setups slightly differ in metal support geometry and only IMRS uses oil immersion.

Binary polymer solutions were prepared from poly (vinyl acetate) (PVAc, Carl Roth, 9154.1) and methanol (MeOH, Carl Roth, 4627.1) by weight with an initial solvent load of $X_{0}=1$, 1.5, and $2\;g_{\mathrm{MeOH}}/g_{\mathrm{PVAc}}$. The solutions were mixed on a roll mixer at ambient temperature for at least one week. Films were blade coated with custom coaters and coating gaps of $h_{gap}=50$, 100, 150, and 200 μm on microscope-grade borosilicate glass substrates with a thickness of approximately 150 μm. The films had an approximate extent of 5–8 cm in coating direction and 2 cm in cross-coating direction, respectively. The glass substrates were mechanically attached on top of a hollow metallic support, temperature-controlled by a water thermostat set to $T_{substrate}=20\;{}^{\circ}C$. The metallic support has an opening in the center for optical access to the film from below, which is necessary for transient microscopic flow-field measurements reported in Reference [1], as well as concentration measurements during drying in this study, detailed in Section 2.2. The opening is also temperature-controlled from below with a temperature-controlled airflow around the tip of the microscope lenses (see Figure 2). Drying of the films was performed under ambient conditions. In order to mitigate the effect of lab ventilation airflow on drying, the films were covered with a box of approximately 15 cm edge length with open top. The same conditions were used for the previously published 3D-µPTV experiments [1]. The experimental conditions are summarized in Table 1.

### 2.2. Transient 1D Concentration Measurements

Inverse Micro Raman Spectroscopy (IMRS), a measurement technique developed in our group [4,23,24], was used to measure transient vertical concentration profiles in the drying PVAc-methanol films. The optical access to the film is realized by an inverse microscope positioned below and directed upward, measuring through a glass substrate (Figure 2a). This is identical to the previously used 3D-µPTV (Figure 2b) [1]. Vertical scanning with a piezo actuator allows for measurements of transient concentration profiles over the film height. In order to grant a high spatial resolution (≈1 μm), immersion oil between microscope lens and glass substrate is necessary. This is a relevant difference compared to previously used 3D-µPTV where no immersion liquid was used, as it slightly affects the temperature control of the glass substrate above the lens opening. The experimental conditions are summarized in Table 1. Similar to 3D-µPTV, the refractive index of the drying polymer film is needed in order to correctly assess the vertical measurement position. Additional details regarding the setup and evaluation can be found in References [4,23].

### 2.3. Temperature Measurements

In order to assess the lateral uniformity of heat control of the substrate (see Figure 2), drying experiments were performed ($X_{0}=1$ and $2\;g_{\mathrm{MeOH}}/g_{\mathrm{PVAc}}$, $h_{gap}=200\;\mu m$) on temperature-controlled glass substrates, coated with black spray varnish prior to experiments. A thermal imaging camera (FLIR, T530) was used to measure the lateral temperature distribution and the black coating acts as a common reference background. Hence, simultaneous flow field or concentration measurements are not possible. Due to physical constraints, these experiments were conducted without the covering box, intended to minimize the impact of lab ventilation on drying.

### 2.4. Film Drying Simulations

Based on experimental IMRS findings, an isothermal one-dimensional film drying simulation model has been developed in the past. It accounts for vertical Fickian solvent diffusion in the film, the concentration-dependent phase-equilibrium of polymer-solvent solutions at the free surface and a constant mass transfer coefficient $\beta_{solvent,air}$ in the gas phase above the film. Since β is a solvent-specific value, it is established to state the material-independent heat transfer coefficient $\alpha_{top}$ instead (linked to β by Lewis law, e.g., Reference [77]). The isothermal model was validated for many different binary polymer-solvent combinations in the past, including PVAc-methanol [4,5,11]. In this work, the lower boundary condition is a zero flux condition, since the glass substrate is impermeable. The drying air is assumed free of solvent.

An extension accounting for non-isothermal drying conditions including up to three substrate layers was published in Reference [29]. Since vertical temperature profile measurements in thin drying films with a thickness in the order of micrometers is a challenging task yet to be resolved, the vertical temperature profiles of the non-isothermal simulation model has not been validated quantitatively. However, a different implementation of the same governing equations shows good agreement with temperatures measured at the bottom side of the substrate [78].

In this work, we use the simulation model in two ways: First, as an elaborated fit to experimental IMRS results in order to assess whether the isothermal 1D model can account for vertical concentration profiles in films with 3D short-scale convective instabilities during drying. The dry film thickness was fixed to experimental results. The solvent content in the surrounding air was fixed to $y_{\infty }=0$ and $\alpha_{top}$ was varied in steps of 0.1 W/ (m^2^ K) until a best match between local and integral concentration profiles between experiment and simulation was found. We term the resulting heat transfer coefficient effective, since it might account for accumulation of methanol vapor in the air above the film due to lack of forced convective airflow. The true $\alpha_{top}$ might be larger.

Second, we perform non-isothermal simulations matching the experimental conditions from previously reported 3D-µPTV measurements at the lens opening, in order to access vertical temperature profiles and to calculate transient thermal and solutal Marangoni numbers, respectively. The boundary temperature $T_{\infty }=22.5\;{}^{\circ}C$ was fixed to lab temperature conditions below the substrate and above the film. We account for the glass substrate and an unknown $\alpha_{bottom}$ due to the forced convective airflow around the microscope lens for substrate temperature control. Therefore, we performed a parameter variation with $\alpha_{bottom}=25$, 50, and $150\;W/\left(m^{2}K\right)$. For each value of $\alpha_{bottom}$, the upper effective heat-transfer coefficient $\alpha_{top}$, matching the previously published 3D-µPTV experiments, was found by varying $\alpha_{top}$ as described for the isothermal simulations until the transient film height best matched the experimental results.

### 2.5. Material Properties

Several material properties of the used PVAc-methanol solutions are required for experimental evaluation, as well as simulation and Marangoni number calculation: The temperature- and concentration-dependent surface tension σ, being the driving force of Marangoni instabilities, is crucial for a realistic assessment of stability thresholds. For poly(vinyl acetate)-methanol, the surface tension increases from $\sigma_{MeOH}\left(20\;{}^{\circ}C\right)=22.6\;\mathrm{mN}/m$ for pure methanol to $\sigma_{PVAc}\left(20\;{}^{\circ}C\right)=36.5\;\mathrm{mN}/m$ for pure PVAc [44]. As for most materials, σ linearly decreases with increasing temperature, e.g., Reference [79], with $\partial \sigma /\partial T=-0.090\pm 0.007\;\mathrm{mN}/\left(m\cdot K\right)$ between 10 °C and 40 °C [44]. The concentration dependency, however, is non-linear: Between a methanol mass fraction of $x_{MeOH}=0.60$ to 0.67, the partial derivative is $\partial \sigma /\partial x_{MeOH}\approx -7.6\;\mathrm{mN}/m$, and, between $x_{MeOH}=0.43$ to 0.50, it is $\partial \sigma /\partial x_{MeOH}\approx -18.0\;\mathrm{mN}/m$ [44]. This shows that the assumption of linear concentration dependency, as assumed in most theoretic analyses, is not valid for our experiments.

Experimental values of the viscosity, known to strongly increase with decreasing solvent content, and an exponential fit were reported in Reference [1] for a temperature range of 10–40 °C and a methanol concentration of 38–100 wt%. The glass transition temperature of pure PVAc is between 29–33 °C [4], exceeding the drying temperature used in this study and during the previously reported 3D-µPTV experiments (≈20 °C). With a small addition of 4 wt% methanol, the glass transition temperature decreases below 15 °C [4].

The Flory-Huggins interaction parameter for calculating the phase equilibrium, as well as the binary diffusion coefficient of poly (vinyl acetate)-methanol, have been taken from References [4,5], respectively. The diffusion coefficient $D\left(20\;{}^{\circ}C\right)$ for diluted solutions is in the order of 10^9^ m^2^/s, decreasing strongly with decreasing methanol content to a limiting value in the order of 10^13^ m^2^/s for pure poly (vinyl acetate).

The remaining properties required to describe the coating solution are density ρ, refractive index n, heat conductivity λ, and heat capacity $c_{p}$. These parameters do not vary much with concentration. Therefore, they are calculated with ideal mixing rules from pure component data [77]. For pure methanol, additional properties as input for the simulation are the vapor pressure $p^{*}$ and the heat of vaporization $\Delta h_{v}$. Data for pure methanol and poly (vinyl acetate) are taken from References [77,80,81,82], respectively. The thermal properties of the glass substrate are taken from manufacturer data [83,84]. All values and applied mixing rules are given in Appendix A Table A1, Table A2, Table A3, Table A4 and Table A5.

### 2.6. Calculation of Marangoni Numbers

The general idea of the Marangoni number Ma is that the driving force for convection, namely the surface tension gradient, is represented in the numerator, while balancing forces are given in the denominator. The surface tension depends on temperature and concentration; hence, gradients in temperature and concentration may give rise to a surface tension gradient. Consequently, the thermal diffusivity a, as well as the diffusion coefficient D, represent the ability to mitigate these gradients and are given in the denominator, along with the liquid viscosity η. The thermal Marangoni number $Ma_{T}$ was calculated as

(1) $$Ma_{T}=\Delta \sigma \left(\bar{x},T\right)\;\times \;\frac{h}{\eta \left(\bar{x},\bar{T}\right)\times a\left(\bar{x},\bar{T}\right)}\;$$

with $\Delta \sigma \left(\bar{x},T\right)$ being the surface tension difference between surface and bottom of the film, subject to local temperature but with height-averaged concentration. The bars above symbols indicate values averaged over the film height. The solutal Marangoni number $Ma_{s}$ was defined accordingly as

(2) $$Ma_{s}=\Delta \sigma \left(x,\bar{T}\right)\times \;\frac{h}{\eta \left(\bar{x},\bar{T}\right)\times D\left(\bar{x},\bar{T}\right)}\;$$

The subscript of Ma denotes whether the instability is driven by thermal or solutal effects. These definitions are equivalent to previously published works. Since past publications do not account for η, a, and D variations over the film height due to drying-induced vertical concentration and temperature gradients, we additionally calculate Ma using not height-averaged values as in Equations (1) and (2) ($\bar{x}$, $\bar{T}$), but using local values for x and T at the bottom (e.g., $\eta \left(x_{bottom},T_{bottom}\right)$) and surface (e.g., $\eta \left(x_{surface},T_{surface}\right)$) of the film for all properties in the denominators, respectively. This way the impact of the choice of transient reference temperature and concentration on the magnitude of the Marangoni numbers can be assessed. Note that the vertical surface tension difference Δσ is occasionally given as $\partial \sigma /\partial R\cdot {\Delta R}_{\mathrm{vertical}}$, with R being either temperature or concentration.

## 3. Results

The results are structured as follows: Initially, the vertical concentration profiles from 1D drying simulations are validated with IMRS experiments (Section 3.1). The comparability of simulations with 3D-µPTV experiments is discussed in Section 3.2. In Section 3.3, we report results from non-isothermal drying simulations under variation of $\alpha_{bottom}$. Finally, by combining the previously reported 3D-µPTV and non-isothermal simulation results, a stability-threshold assessment based on realistic transient Marangoni numbers is made in Section 3.4.

### 3.1. Validation of Simulated Vertical Concentration Profiles

In order to gain experimental access to transient concentration profiles for further analysis of the previously reported 3D-µPTV experiments, the same film drying experiments were conducted on the IMRS measurement setup with close to identical drying conditions with the sole systematic difference being the immersion medium. Subsequently, 1D isothermal drying simulations were matched to the experimental results by varying the upper heat transfer coefficient in steps of $0.1\;W/\left(m^{2}K\right)$, as described in Section 2.4.

**Figure 3:**
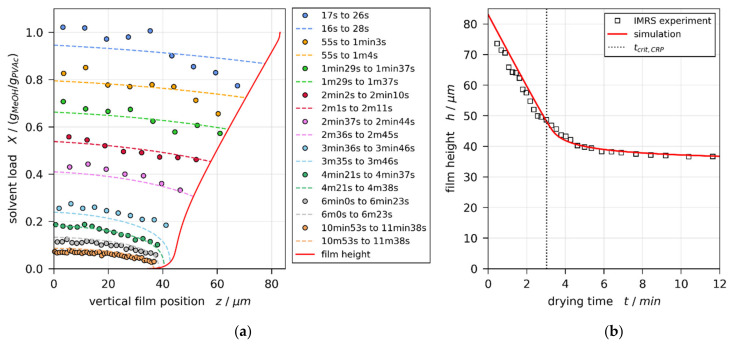
Comparison of experimental IMRS and best-matching simulation results for a drying poly (vinyl acetate)-methanol film ($X_{0}=1\;g_{\mathrm{MeOH}}/g_{\mathrm{PVAc}}$ and $h_{gap}=200\;\mu m$, convectively stable according to Reference [1]), drying at $T_{\mathrm{substrate}=20\;℃}$ and otherwise ambient conditions. The simulation was performed with experimental dry-film thickness and initial solvent load, as well as an effective upper heat-transfer coefficient $\alpha_{\mathrm{top}}$=1.4 W/($m^{2}$·K). (**a**) Vertical solvent-load profiles at different drying times. Since IMRS is a scanning method, the acquisition time is given in ranges. This has been accounted for in the simulation. (**b**) Transient film height (drying curve) and end of constant rate period $t_{\mathrm{crit}},P$.

Figure 3a,b show the IMRS derived transient one-dimensional vertical concentration profiles (colored round markers) and drying curve (transient film height, black squares), respectively, for a film with initial solvent load of $X_{0}=1\;g_{\mathrm{MeOH}}/g_{\mathrm{PVAc}}$ and coating gap $h_{\mathrm{gap}}=200\;\mu m$ (convectively stable according to previously reported 3D-µPTV experiments [1]). The simulation result best matching the experimental concentration profiles and drying curve is shown in Figure 3, as well. The solid red line denotes the drying curve, while the dashed colored lines denote the vertical concentration profiles. It can be seen that the local concentration profiles, as well as the integral drying curve derived from IMRS experiments, are in reasonable agreement with the simulation results. The vertical black dotted line in Figure 3b denotes the end of the constant rate period $t_{crit,CRP}$.

**Figure 4:**
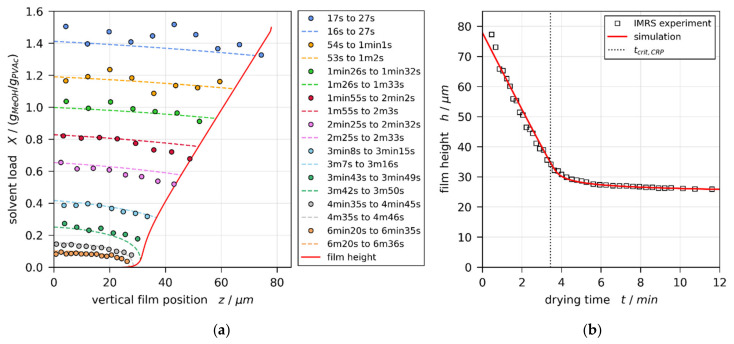
Comparison of experimental IMRS and best-matching simulation results for a drying poly (vinyl acetate)-methanol film ($X_{0}=1.5{\;g}_{\mathrm{MeOH}}/g_{\mathrm{PVAc}}$ and $h_{gap}=200\;\mu m$, initially convectively unstable until $t_{crit,Ma}=2.4\;\min$ according to Reference [1]), drying at $T_{substrate}=20\;{}^{\circ}C$ and otherwise ambient conditions. The simulation was performed with experimental dry-film thickness and initial solvent load, as well as an effective upper heat-transfer coefficient $\alpha_{top}=1.5\;W/\left(m^{2}\cdot K\right)$. (**a**) Vertical solvent-load profiles at different drying times. Since IMRS is a scanning method, the acquisition time is given in ranges. This has been accounted for in the simulation. (**b**) Transient film height (drying curve) and end of constant rate period $t_{crit,CRP}$.

The same style of plot for a film with initial solvent load of $X_{0}=1.5\;g_{\mathrm{MeOH}}/g_{\mathrm{PVAc}}$ and coating gap $h_{gap}=200\;\mu m$ is given in Figure 4. From previously reported 3D-µPTV results (Figure 1, Reference [1]), it is known that, under these experimental conditions, the film is convectively unstable, exhibiting 3D convection cells until $t_{crit,Ma}=2.4$ min of drying. The vertical transient concentration profiles derived from IMRS experiment show, however, good agreement with simulation results accounting for 1D vertical diffusional mass transport in the film only.

It was found that the vertical concentration profiles, as well as the drying curves from IMRS experiments and simulations, are in good agreement for all combinations of initial solvent load $X_{0}$ and coating gap $h_{gap}$ under consideration in this study, disregarding whether the films exhibited initial convective instabilities, observed during the respective 3D-µPTV experiments reported in Reference [1], or not. This is in line with previously published comparisons of IMRS experiments and 1D simulations of PVAc-methanol films under various (faster) drying conditions [4,5,11,12].

### 3.2. Aspects Regarding the Comparability of µPTV Results with Simulations

In order to derive critical Marangoni numbers, the results from 1D drying simulations and the critical drying time $t_{crit,Ma}$, denoting the end of convective instabilities, measured with 3D-µPTV [1], will be combined. Before that, several aspects regarding the comparability of experiments and simulations have to be considered. Since, with 3D-µPTV, other than with IMRS, no concentration profiles can be measured, the only way to directly compare 3D-µPTV results, IMRS measurements, and simulations is by comparing the transient film thickness. The dry film thickness derived from 3D-µPTV experiments was previously validated, showing good agreement with experimental values from a physical measuring probe with a discrepancy as little as $\Delta h_{dry}=1.0\pm 3.0\;\mu m$ [1].

**Figure 5:**
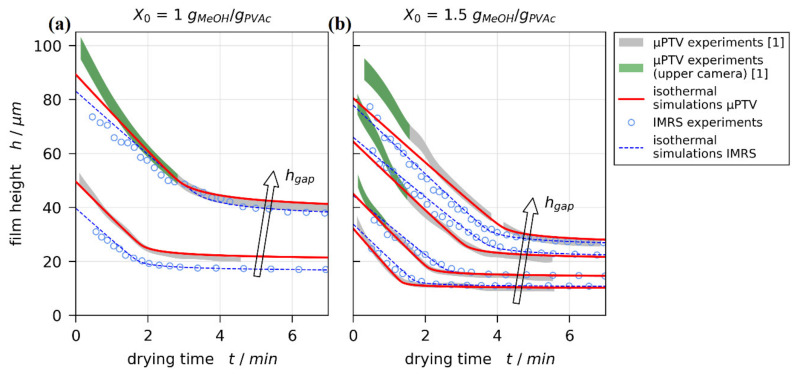
Comparison of drying curves for poly (vinyl acetate)-methanol films at $T_{substrate}=20\;{}^{\circ}C$ and ambient drying. The filled areas (gray-green) denote experimental results from 3D-µPTV measurements accounting for refractive index uncertainty in the evaluation. The green area denotes data recorded with the camera having the highest vertical focus position. Red lines indicate isothermal simulation results with $h_{dry}$ fixed to experimental results. The blue circles and lines denote the experimental IMRS results and matched simulations, respectively. (**a**) $X_{0}=1\;g_{\mathrm{MeOH}}/g_{\mathrm{PVAc}}$, $h_{gap}=100$ and 200 μm. (**b**)$\;X_{0}=1.5\;g_{\mathrm{MeOH}}/g_{\mathrm{PVAc}}$, $h_{gap}=50$, 100, 150, and 200 μm.

The experimental 3D-µPTV drying curves from films with initial solvent load of $X_{0}=1$ and $1.5\;g_{\mathrm{MeOH}}/g_{\mathrm{PVAc}}$ are given in Figure 5 as gray-green filled areas. Due to the refractive index dependency of the vertical tracer particle position calibration [75], the true drying curve should coincide with the lower bounds of the filled area at t=0 s and with the upper bounds for solvent depleted films (see black curves in Figure 1 and Reference [1]). Isothermal simulations with dry film thickness pinned to experimental results were matched to the experimental drying curves by varying the upper effective heat transfer coefficient $\alpha_{top}$ in steps of $0.1\;W/\left(m^{2}K\right)$. The simulations best matching the gray uncertainty areas of 3D-µPTV experiments are given in Figure 5 as red lines. Additionally, the experimental IMRS results (blue markers) and matched simulations (blue lines) are depicted.

The 3D-µPTV simulation (red lines) and IMRS results (blue markers and lines) are in reasonable agreement. While the transient course of drying curves might be influenced by convective instabilities during drying, the initial wet film thickness $h_{0}$ (t=0 s) is solely governed by the blade coating process. It is known that $h_{0}$ can be affected by coating velocity and solution viscosity [85]. Since our films were coated with identical but manually operated blade coaters, small variations in $h_{0}$ are to be expected. This appears more pronounced for the initial solvent load $X_{0}=1{\;g}_{\mathrm{MeOH}}/g_{\mathrm{PVAc}}$, having a larger initial viscosity, while $h_{0}$ aligns almost perfectly for an initial solvent load of $1.5{\;g}_{\mathrm{MeOH}}/g_{\mathrm{PVAc}}$. Due to the 1D approach of the drying simulations, this also impacts the film height in the diffusion-controlled drying regime (plateau after constant rate period).

Comparing the 3D-µPTV experimental drying curves (filled areas) with the respective matched simulations (red lines), they are in excellent agreement in the diffusion-controlled regime and large portions of the constant rate period. There is, however, an initial noticeable systematic discrepancy between 3D-µPTV experiments and 1D simulations for thicker films. This discrepancy almost exclusively occurs when the experimental film height was derived from data of the camera with the highest vertical focus position, shown in Figure 5 as green areas and in Figure 1 as green trajectories. This discrepancy is not observable for IMRS experiments and simulations.

The sole systematic difference between the 3D-µPTV and IMRS experimental setup is the substrate temperature control at the lens opening because IMRS requires immersion oil between the microscope lens and the glass substrate, whereas 3D-µPTV requires no immersion (air gap) between lens and substrate. Hence, two possible explanations can account for the observed discrepancy between experimental and simulated 3D-µPTV results: Either the previously experimentally derived drying curves are correct, which would indicate that there is an additional lateral flow away from the lens opening due to an imperfect lateral temperature control associated with the opening, which the 1D simulation cannot account for. Alternatively, there is a systematic flaw in the vertical particle position calibration of the upmost camera, which has yet to be resolved.

**Figure 6:**
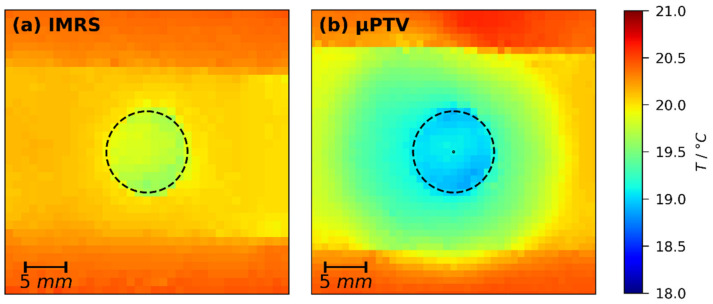
Lateral temperature of poly (vinyl acetate)-methanol films ($X_{0}=2{\;g}_{\mathrm{MeOH}}/g_{\mathrm{PVAc}}$, $h_{gap}=200\;\mu m$) with substrate set temperature $T_{substrate}=20\;{}^{\circ}C$ at t=20 s drying time (constant rate period), measured with a thermal imaging camera. (**a**) IMES; (**b**) μPTV. The noticeable horizontal stripes are the films and the black dashed circle indicates the lens opening in the metal support. The tiny black circle in (**b**) represents the lateral extent of the 3D-µPTV field-of-view.

In order to assess the impact of the lens opening in the temperature-controlled substrate support, especially the impact of the immersion medium, lateral temperature measurements in additional representative film drying experiments were conducted as described in Section 2.3. Figure 6 shows exemplarily thermal images of drying films ($X_{0}=2\;g_{\mathrm{MeOH}}/g_{\mathrm{PVAc}}$, $h_{gap}=200\;\mu m$) using the temperature-controlled substrate of IMRS (a) and 3D-µPTV (b) setup at t=20 s drying time (during constant rate period). The noticeable horizontal stripe in the images is the drying film and the dashed circles indicate the lens openings of the respective substrate supports necessary for optical access for both measurement techniques. It can be seen that for both setups there is a lateral temperature difference between the film above the opening and above the solid support. However, due to the slightly different geometry of the opening and the absence of immersion oil during 3D-µPTV experiments, the lateral temperature difference differs slightly. Raman experiments (Figure 6a) exhibit $\Delta T_{lateral}=T_{solid}-T_{opening}\approx 0.3\pm 0.2\;{}^{\circ}C$ and 3D-µPTV experiments exhibit $\Delta T_{lateral}\approx 1.2\pm 0.1\;{}^{\circ}C$. We have tried to mitigate this small but measurable discrepancy by increasing the set temperature of the airflow in the opening of the 3D-µPTV substrate support. It turned out that, with the current geometry of the 3D-µPTV support, solid substrate temperature and opening temperature cannot be controlled independently, which will be improved in future work.

From drying experiments with an aluminum support having a partial Teflon inlay, reported in Reference [68], we know that, in this case, thermal and solutal lateral long-scale Marangoni forces act in opposite directions: Considering only thermal effects on surface tension, a cooler film above the Teflon inlay (or, in this work, the opening) gives rise to an increased surface tension compared to its lateral surrounding. This would imply a lateral long-scale Marangoni flow towards the opening. Considering only solutal effects on surface tension, a cooler film above the Teflon inlay (or the opening, in this work) would retard the drying rate due to a decrease in methanol vapor pressure. This gives rise to a higher methanol concentration in the film above the opening. Since the surface tension of poly (vinyl acetate)-methanol solutions is decreasing with increasing methanol concentration (see Section 2.5), the solutal effect would imply a lower surface tension above the opening and, therefore, a lateral long-scale Marangoni flow away from the opening. Results from Reference [68] show that the solutal effect is dominant for $X_{0}=2\;g_{\mathrm{MeOH}}/g_{\mathrm{PVAc}}$, $T_{substrate}=30\;{}^{\circ}C$, and a lateral temperature difference of $\Delta T_{lateral}\approx 10\;{}^{\circ}C$.

The good agreement between experimental IMRS results and the 1D vertical simulations presented in Section 3.1 indicate that there is no such long-scale lateral flow in IMRS experiments with a lateral temperature difference of $\Delta T_{lateral}\approx 0.3\pm 0.2\;{}^{\circ}C$. For the 3D-µPTV drying experiments, the lateral temperature difference was found to be slightly larger. This means that there is the remote possibility that the previously reported 3D-µPTV drying experiments may exhibit a long-scale lateral Marangoni flow away from the lens opening, which may break up into the observed short-scale vertical convection cells due to the radial nature of the lateral temperature difference [1]. In the following, we present three reasons why we believe this not to be the case:

First, the discrepancy between experimental and simulated drying curves also occurs for $X_{0}=1\;g_{\mathrm{MeOH}}/g_{\mathrm{PVAc}}$ and $h_{gap}=200\;\mu m$ (Figure 5a, upper curve). The flow field of this experiment reported in Reference [1], however, shows only very small initial lateral velocities $u_{lateral}\leq 0.4\;\mu m/s$ and almost no tracer particles exiting the observation volume laterally. For a long-scale lateral Marangoni flow, higher values would be expected (≈60 μm/s in Reference [75]).

Second, Cerisier et al. reported experimental findings on thermally-induced short-scale Marangoni convection cells in films of pure liquids with a focus on the aspect ratio $\Gamma =d_{lateral}/h$, with $d_{lateral}$ being the lateral extent of the films [86]. They found that the lateral cell size increased with increasing aspect ratio up to Γ≤70. For higher aspect ratios, the lateral cell size was constant. The authors conclude that films with Γ>70 can be treated as infinitely extended, unaffected by the lateral bounds. The experiments reported in this work have an initial wet-film thickness of $h_{0}\approx 20$ to 100 μm, decreasing during drying. With a lateral extent of the lens opening $d_{lateral}=10\;\mathrm{mm}$, this translates to initial aspect ratios Γ≥100 to 500, increasing during drying. Therefore, it is safe to assume that the lateral temperature difference due to the lens opening does not affect the flow field above the center of the opening (see Figure 6b for size-comparison).

**Figure 7:**
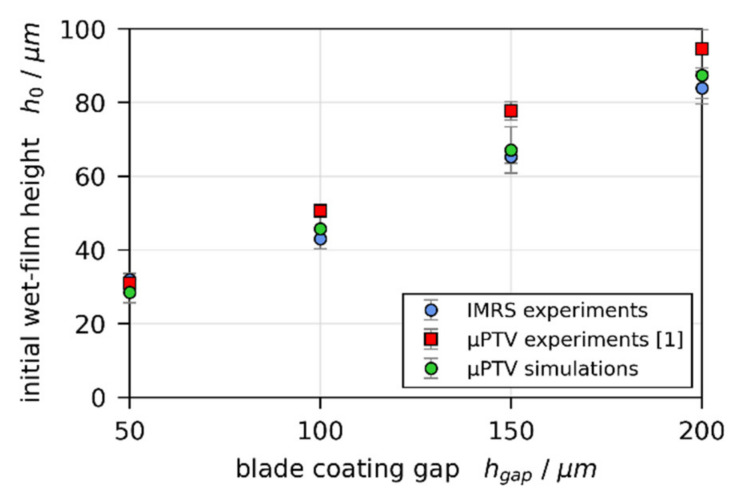
Comparison of initial wet-film thickness $h_{0}$ extrapolated to t=0 s from experimental data of 3D-µPTV (red markers) and IMRS (blue markers), as well as from 1D simulations, matched to the dry-film thickness of 3D-µPTV experiments (green markers).

Third, the initial wet film thickness $h_{0}$ is solely governed by the blade coating process. However, comparing $h_{0}$ extrapolated from the constant rate period to t=0 s for all IMRS and previously reported 3D-µPTV experiments, a systematic discrepancy can be observed (Figure 7). The initial wet film thickness extracted from the 3D-µPTV experiments (red markers) is systematically larger than $h_{0}$ from IMRS experiments (blue markers), while $h_{0}$ derived from 1D-simulations, fitted to the dry film thickness of 3D-µPTV experiments (green markers), are in good agreement with the IMRS results. If a long-scale lateral Marangoni flow would be present, a thinner initial wet film thickness of the 3D-µPTV matched 1D simulations, compared to the IMRS results, would be expected (at least for a dominating solutal influence), since polymer would have been dragged away from the center of the film as stated above.

In light of these facts, we are confident that the previously reported 3D-µPTV experiments were not affected by lateral long-scale convection induced by the small lateral temperature difference above the solid substrate and the lens opening. Instead, a yet to be resolved issue in vertical particle-position calibration of the upmost camera seems to cause the observed initial discrepancy between 3D-µPTV derived and simulated drying curves in Figure 5. Based on this argumentation, we are confident that the matched simulations are a good quantitative representation of the previously reported experiments.

### 3.3. Non-Isothermal Simulations

Up to this point, all previously reported simulation results were isothermal. In order to assess thermal effects on Marangoni instabilities, non-isothermal drying simulations matching 3D-µPTV experiments were conducted, as well. The non-isothermal 1D model accounts for evaporative cooling of the drying film surface, the 1D heat conduction in the glass substrate and the temperature-controlled airflow in the lens opening below the glass substrate (see Figure 2). Due to the complex geometry at the opening, the heat transfer coefficient below the glass substrate $\alpha_{bottom}$ is unknown. Therefore, the non-isothermal drying simulations were performed with parameter variation of $\alpha_{bottom}$, as described in Section 2.4.

**Figure 8:**
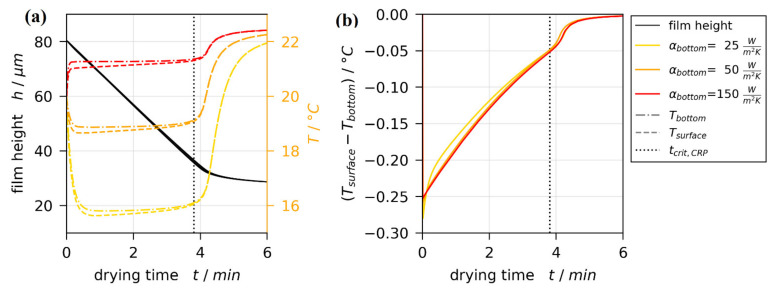
Results from a non-isothermal 1D drying simulation with $X_{0}=1.5{\;g}_{\mathrm{MeOH}}/g_{\mathrm{PVAc}}$, $h_{dry}=25\;\mu m$, and $\alpha_{bottom}$ variation. The dotted vertical line indicates the end of the constant rate period $t_{crit,CRP}$. (**a**) Drying curves and temperatures at bottom and surface of the drying film. (**b**) Vertical temperature difference between surface and bottom of the film.

Figure 8 shows exemplarily the non-isothermal simulation results for a single set of boundary conditions. The black line in Figure 8a denotes the drying curves of an isothermal simulation, as well as the drying curves of three non-isothermal simulations with $\alpha_{bottom}$ variation. The black lines coincide perfectly, as intended. Therefore, the argumentation regarding drying curve comparison presented in the last section remains unaffected. The transient temperatures at the bottom (dash-dotted colored lines) and the surface (dashed colored lines) of the film, however, differ noticeably with $\alpha_{bottom}$. While all simulation results show a close to constant temperature during the constant rate period ($t\leq t_{crit,CRP}$), this steady-state temperature increases with increasing heat transfer coefficient at the bottom. For the three values investigated, the simulated film temperature for $\alpha_{bottom}=50\;W/\left(m^{2}K\right)$ is closest to the temperatures measured with thermal imaging shown in Figure 6b within the dashed circle.

In regard to short-scale Marangoni instabilities, the temperature difference between top and bottom of the film is of significant importance. Figure 8b shows that this difference is very similar for all values of $\alpha_{bottom}$, indicating that the exact value thereof is of minor importance. These results hold true for all non-isothermal simulations.

### 3.4. Marangoni Stability Threshold

From the previously published 3D-µPTV experiments, it was found that some films are convectively stable, while others initially exhibit short-scale Bénard-Marangoni convection cells, but become stable at a critical drying time $t_{crit,Ma}$ during the constant rate period [1]. In Section 3.1, we validated the local concentrations from the 1D simulation model with IMRS experiments. In Section 3.2, we established that the simulated drying curves are in agreement with previously reported experimental 3D-µPTV results, and, in Section 3.3, we presented that $\alpha_{bottom}$ variation has little impact on the vertical temperature difference in the film. We can now combine the information regarding convective (in)stability from 3D-µPTV experiments ($t_{crit,Ma}$) with transient local concentrations, temperatures, and film height from the non-isothermal simulations.

#### 3.4.1. Critical Initial Wet Film Thickness

**Figure 9:**
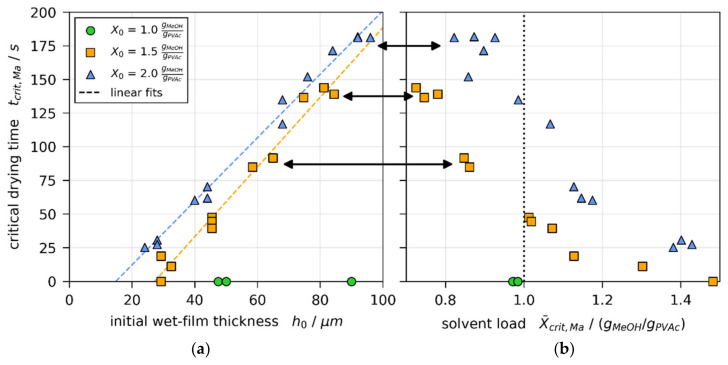
Critical drying time $t_{crit,Ma}$, denoting the transition from convectively unstable to stable during 3D-µPTV drying experiments [1]. (**a**) Plotted over initial wet film thickness $h_{0}$ from matched simulations. *Modified from [1], License: CC-BY 4.0.* (**b**) Plotted over height-averaged solvent load ${\bar{X}}_{crit,Ma}\left(t_{crit,Ma}\right)$ from matched simulations. The black arrows denote experiment groups where ${\bar{X}}_{crit,Ma}<1\;g_{\mathrm{MeOH}}/g_{\mathrm{PVAc}}$, although experiments with initial solvent load $X_{0}=1\;g_{\mathrm{MeOH}}/g_{\mathrm{PVAc}}$ were convectively stable from the start of drying [1].

Figure 9a shows the critical drying times $t_{crit,Ma}$, at which initially convectively unstable films become stable, over the initial wet film thickness for all 3D-µPTV drying experiments. While all films with $X_{0}=1\;g_{\mathrm{MeOH}}/g_{\mathrm{PVAc}}$ were initially stable ($t_{crit,Ma}=0\;s$, green markers), the critical drying time decreases linearly with decreasing $h_{0}$ for $X_{0}=1.5$ and $2\;g_{\mathrm{MeOH}}/g_{\mathrm{PVAc}}$ (orange and blue markers), respectively. The linear fits (dashed lines) intersect with the *x*-axis at $h\left(X_{0}=1.5\;g_{\mathrm{MeOH}}/g_{\mathrm{PVAc}}\;\right)=27.1\;\mu m$ and $h\left(X_{0}=2\;g_{\mathrm{MeOH}}/g_{\mathrm{PVAc}}\;\right)=14.8\;\mu m$, respectively. This implies that there is a critical film thickness, varying with $X_{0}$, below which films stay convectively stable. Figure 9b shows the height-averaged solvent load at $t_{crit,Ma}$ from the matched non-isothermal simulations. It can be seen that initially unstable thicker films, indicated by black arrows, become stable at a height-averaged solvent load well below $\bar{X}=1\;g_{\mathrm{MeOH}}/g_{\mathrm{PVAc}}$ (vertical dotted line), even though films with initial solvent load of $X_{0}=1\;g_{\mathrm{MeOH}}/g_{\mathrm{PVAc}}$ were entirely stable. These findings indicate a hysteresis regarding the solvent load at the onset and termination of convective instabilities. The lowest local solvent load at $t_{crit,Ma}$ was found to be $\geq 0.7{\;g}_{\mathrm{MeOH}}/g_{\mathrm{PVAc}}$ (41 wt% methanol). At this concentration, the glass transition temperature is significantly below the drying temperature (see Section 2.5). Hence, a possible glass transition at the surface does not cause the convective instabilities to stop.

#### 3.4.2. Transient Marangoni Numbers during Drying

**Figure 10:**
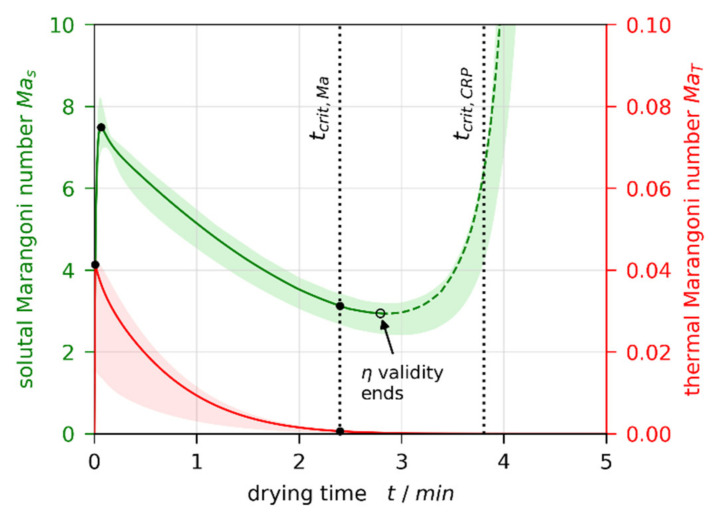
Transient thermal and solutal Marangoni numbers calculated from a non-isothermal simulation matched to a µPTV experiment ($X_{0}=1.5\;g_{\mathrm{MeOH}}/g_{\mathrm{PVAc}}$, $h_{dry}=25\;\mu m$, $\alpha_{bottom}=50\;W/\left(m^{2}K\right)$). $Ma_{s}$ is two orders of magnitude larger than $Ma_{T}$. The colored areas indicate the uncertainty due to $\alpha_{bottom}$ variation, as well as choice of reference concentration and temperature for η, a, and D calculation. The black filled circles indicate extracted data for further discussion. The dashed portion of $Ma_{s}$ is deemed invalid, since viscosity data are extrapolated and the film starts to solidify.

**Figure A1:**
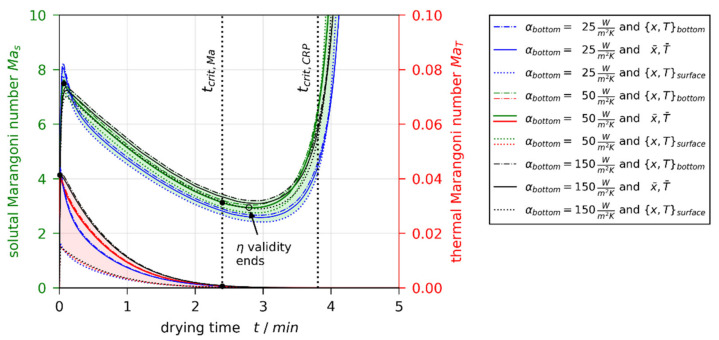
Same as Figure 10, but with all individual results summarized in the colored uncertainty areas.

From the simulation results matched to 3D-µPTV experiments, we calculate transient thermal and solutal Marangoni numbers $Ma_{T}$ and $Ma_{s}$, respectively, as defined in Section 2.6. Figure 10 shows exemplarily the results for $X_{0}=1.5\;g_{\mathrm{MeOH}}/g_{\mathrm{PVAc}}$, $h_{dry}=25\;\mu m$, and $\alpha_{bottom}=50\;W/\left(m^{2}K\right)$ (red and green lines), which are qualitatively identical for all simulations. The colored areas indicate the uncertainty, accounting for $\alpha_{bottom}$ variation, as well as calculating the material properties in the denominator of the Marangoni numbers (viscosity, thermal diffusivity, and diffusion coefficient) not with height-averaged concentration and temperature, but evaluated at the surface and the bottom of the film, respectively. Hence, the colored areas comprise results of nine individual parameter sets. The individual curves are provided in Appendix B Figure A1.

Both curves show a maximum at the beginning of drying (t≈0), initially decreasing during drying. While the thermal Marangoni number asymptotically decreases towards zero, the solutal Marangoni number strongly increases nearing the end of the constant rate period (CRP). This is because a solvent-depleted surface layer forms (see strongly curved concentration profiles near the film surface in Figure 3a and Figure 4a), which increases the surface tension difference Δσ between surface and bottom of the film. In addition, the diffusion coefficient, being in the denominator of $Ma_{s}$, strongly decreases for low solvent concentrations. At this stage, however, the film is almost dry and solidified. Therefore, no further convective instability can occur. In addition, the validity of the viscosity fit from Reference [1] (see Table A5 in Appendix A) ends before the end of the constant rate period is reached (black empty circle). Consequently, the dashed part of the $Ma_{s}$ curve is disregarded. The transition from convectively unstable to stable ($t_{crit,Ma}$) occurs during the valid portion of the curve. This holds true for all simulations. The black dots highlight values at the initial maximum for all films, as well as at the stability threshold for initially convectively unstable films, extracted for the following discussion.

#### 3.4.3. Critical Marangoni Number

Table 2 summarizes all calculated properties, grouped with regard to initial (in)stability (t≈0, first two data columns) and at the stability threshold $t_{crit,Ma}$ for initially unstable films (last data column). First, it has to be noted that the temperature, as well as solvent load difference, between surface and bottom of the films are very small, due to the small film thicknesses and very mild drying conditions. Second, the calculated values for thermal and solutal Marangoni number are significantly smaller than theoretically derived values found in past work. This is most likely because most available predictive models assume low viscous pure liquids or diluted solutions and additional simplifications, summarized in Section 1.3. The extracted values of the diffusion coefficient, for example, vary by a factor of approximately three. In light of the fact that D changes several orders of magnitude with methanol concentration, the extent of D appears small. However, it is far from constant, as assumed in past theoretic work.

Comparing the thermal and solutal Marangoni numbers (Table 2, last row) reveals that $Ma_{T}$ is two orders of magnitude smaller than $Ma_{s}$, which indicates that the solutal effect is dominant. Consequently, we limit the following discussion on concentration-gradient-induced Marangoni instabilities only. When comparing the solutal Marangoni numbers $Ma_{s}$ at the onset of drying (t≈0, Table 2 first two columns), there is no distinction in value range between convectively stable films ($1.1\leq Ma_{s}\leq 10.3$) and initially unstable ones ($0.8\leq Ma_{s}\leq 11.5$). There is, however, a noticeable difference in Schmidt numbers with only little value overlap ($0.7\cdot 10^{6}\leq Sc\leq 6.6\cdot 10^{6}$ and $0.2\cdot 10^{6}\leq Sc\leq 0.8\cdot 10^{6}$, respectively).

**Figure 11:**
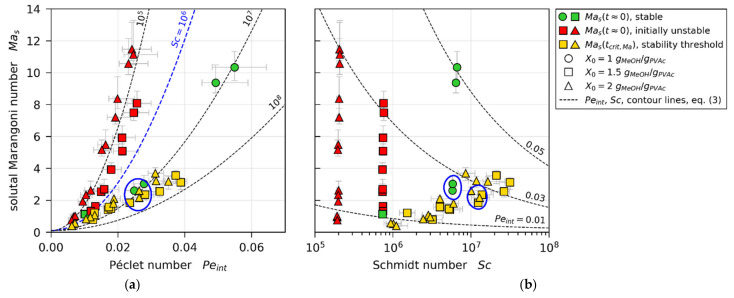
Solutal Marangoni numbers extracted from drying simulations matched to µPTV experiments. (**a**) Plotted over Péclet number. (**b**) Plotted over Schmidt number.

In line with theoretically found trends from Trouette et al. (see Section 1.3), Figure 11 shows all $Ma_{s}$ values from our 3D-µPTV-matched simulations plotted over Péclet number (Figure 11a) and Schmidt number (Figure 11b), respectively. With regard to $Pe_{int}$, it can be seen that there is a clear distinction between values of initially unstable films ($Ma_{s}\left(t\approx 0\right)$, red markers), and $Ma_{s}\left(t\approx 0\right)$ values of stable films, as well as values at the stability threshold (green and yellow markers). When accounting for an increase of critical Marangoni number $Ma_{s,crit}$ with increasing $Pe_{int}$ (blue dashed curve), the instable (red) values reside above $Ma_{s,crit}\left(Pe_{int}\right)$, while most stable and threshold values reside below (green and yellow). There is, however, little difference to be seen between some initially stable films ($Ma_{s}\left(t\approx 0\right)$, green markers) and values at the stability threshold of initially unstable films ($Ma_{s}\left(t_{crit,Ma}\right)$, yellow markers), highlighted with a blue circle. Figure 11b shows the same $Ma_{s}$ values plotted over Sc. It can be seen that the non-distinguishable values with regard to $Pe_{int}$, highlighted with a blue circle, have noticeably different Schmidt numbers.

In order to account for the dependency of critical solutal Marangoni number on $Pe_{int}$, as well as Sc, we propose a simple empiric power-law relation,

(3) $$Ma_{s,crit}=7.5\times 10^{5}\times Pe_{int}^{2}\times Sc^{-1/3}+0.1\;$$

**Figure 12:**
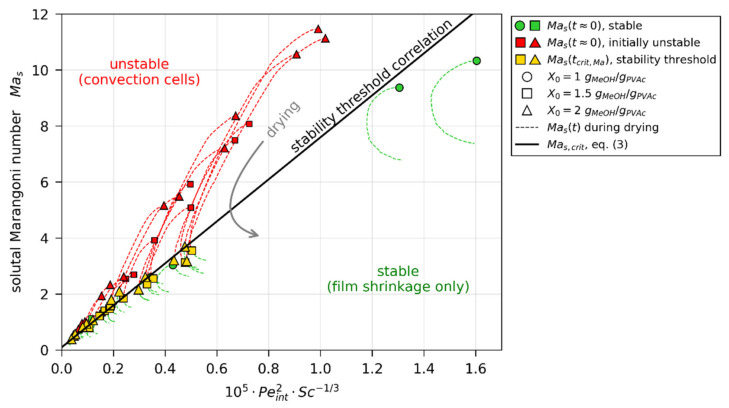
Solutal Marangoni numbers, calculated from simulations matched to µPTV experiments, as function of condensed parameter comprising Péclet and Schmidt numbers. The dashed lines indicate transient $Ma_{s}$ values for all µPTV experiments, starting at green (stable) or red (initially convectively unstable) markers, respectively.

Contour lines for this correlation are given in Figure 11 as dashed lines. The applicability of the correlation becomes clearer when plotting $Ma_{s}$ over the combined parameter $Pe_{int}^{2}\cdot Sc^{-1/3}$, as done in Figure 12. The $Ma_{s,crit}$ correlation, proposed in Equation (3), then reduces to a straight line. The dashed curves indicate the transient course of the solutal Marangoni number for all 3D-µPTV-matched drying simulations, starting at either a green marker (convectively stable film) or red marker (initially unstable film). While the transient $Ma_{s}$ values of stable films reside entirely below the proposed critical stability threshold, the initially convectively unstable films start with $Ma_{s}$ values above the critical stability threshold and undercut $Ma_{s,crit}$ during the course of drying. The averaged relative deviation between the proposed correlation and the $Ma_{s}\left(t_{crit,Ma}\right)$ values (yellow markers), derived from matching simulations to 3D-µPTV experiments, is 9.0 %. We conclude that the simple empiric power-law correlation (Equation (3)) qualitatively follows trends derived by Trouette et al. [72] from a theoretical stability analysis and can account for the initial convective (in)stability, as well as the transition from unstable to stable during the constant rate period, observed in the previously reported 3D-µPTV drying experiments. Note that the intercept of Equation (3) ($Ma_{s,crit}\left(Pe_{int}^{2}\times Sc^{-1/3}=0\right)=0.1$) is supported by the results. In addition, $Pe_{int}^{2}\times Sc^{-1/3}=0$ would imply that a film with either a film shrinkage velocity of $v_{int}=0\;\mu m/s$ (no evaporation) or an infinitely large viscosity would be exactly at the stability threshold. As this is unlikely to be the case, the introduction of the intercept appears reasonable. It has yet to be assessed whether this simple correlation can account for variations in material system, drying temperature and drying rate. This is, however, not within the scope of this work.

**Figure 13:**
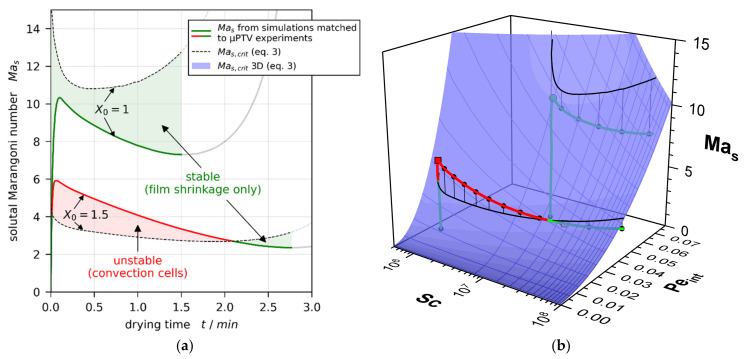
Transient solutal Marangoni number during drying of films with $h_{gap}=200\;\mu m$ and $X_{0}=1$ and $2{\;g}_{\mathrm{MeOH}}/g_{\mathrm{PVAc}}$, respectively, in comparison with the empiric power-law correlation for critical stability threshold. Exemplarily, one convectively stable and one initially unstable film is depicted. (**a**) Plotted over drying time. (**b**) Plotted as function of Péclet and Schmidt numbers. For better visualization, the 3D plot is provided in the Supplementary Material with animated rotation around the vertical axis.

Heretofore, mainly discrete values of $Ma_{s}$ at the start of drying and at the stability threshold of initially unstable films have been discussed in detail. In Figure 13a, the transient course of the solutal (critical) Marangoni number is shown exemplarily for two drying experiments, one being convectively stable and one being initially unstable, becoming stable during the constant rate period. The same results with regard to Péclet and Schmidt numbers are shown in Figure 13b, where the blue surface indicates the $Ma_{s,crit}$ correlation.

The Péclet number $Pe_{int}=v_{int}h/D$ represents the non-dimensional interface velocity, which is a measure for the drying rate, whereas the Schmidt number $Sc=\eta /\left(\rho \cdot D\right)$ describes the ratio of viscous forces to solvent diffusion. Assuming that all other parameters except for the interface velocity $v_{int}$ are constant, the proposed correlation implies that an increase of the drying rate and, therefore, $v_{int}$ results in a higher critical solutal Marangoni value and a more stable film. On the first glance, this appears counterintuitive, since a faster drying rate implies larger vertical concentration gradients, which should make the film less stable. A similar discrepancy can be found when assuming all values to be constant except for the viscosity η. Then, a viscosity increase would imply a larger Sc value, which would imply a smaller critical solutal Marangoni value and a less stable film. However, in reality, a non-trivial coupling between the material properties occurs. Entering the definitions of $Pe_{int}$ and Sc in the proposed correlation Equation (3) results in the proportionality given in Equation (4).

(4) $$Ma_{s,crit}\sim \frac{v_{int}^{2}h^{2}\rho^{1/3}}{\eta^{1/3}D^{5/3}}.$$

Regarding the onset of drying, a decrease of the initial solvent load implies a larger initial viscosity but, simultaneously, a smaller initial diffusion coefficient. Since the diffusion coefficient also appears in the definition of $Pe_{int}$, the coupled concentration-dependent relation $\eta^{1/3}D^{5/3}$ in the denominator of Equation (4) indicates that the reduction of D outweighs the viscosity increase. This ultimately results in a larger critical solutal Marangoni number (more stable film) at the onset of drying, as can be seen by the dashed lines in Figure 13a at t=0 s. During drying, the interplay is even more complex. In Figure 13b, it can be seen that, during drying, the Schmidt number increases because of the simultaneous viscosity increase and diffusion coefficient decrease. The Péclet number $Pe_{int}=v_{int}\cdot h/D$, however, shows a mild local minimum during the constant rate period. While $v_{int}$ is constant during this period, the local minimum indicates that the term h/D is initially dominated by the film shrinkage until the concentration-dependent reduction of the diffusion coefficient outweighs the decrease of h. This results in a non-trivial change of the critical solutal Marangoni number during drying (Figure 13a, black dashed lines; and Figure 13b, black solid surface projections). $Ma_{s}$ of the convectively stable film ($X_{0}=1\;g_{\mathrm{MeOH}}/g_{\mathrm{PVAc}}$) resides below $Ma_{s,crit}$ during the entire drying time, while the initially unstable film ($X_{0}=1.5\;g_{\mathrm{MeOH}}/g_{\mathrm{PVAc}}$) intersects with $Ma_{s,crit}$ and becomes convectively stable during the constant rate period. The transient nature of $Ma_{s,crit}$, therefore, also explains the alleged hysteresis regarding the critical solvent load ${\bar{X}}_{crit,Ma}=\bar{X}\left(t_{crit,Ma}\right)\;<1\;g_{\mathrm{MeOH}}/g_{\mathrm{PVAc}}$ of several initially unstable experiments being smaller than the initially stable films with $X_{0}=1\;g_{\mathrm{MeOH}}/g_{\mathrm{PVAc}}$, reported in Section 3.4.1 (Figure 9).

## 4. Conclusions

In a previously published work, results from drying experiments under ambient conditions with poly (vinyl acetate)-methanol thin films, investigated with 3D micro particle tracking velocimetry (3D-µPTV), have been presented [1]. It was found that films with an initial solvent load of $X_{0}=1\;g_{\mathrm{MeOH}}/g_{\mathrm{PVAc}}$ and an initial wet film thickness of $h_{0}\approx 50$ and 90 μm are convectively stable during the entire course of drying, whereas films with $X_{0}=1.5$ and $2\;g_{\mathrm{MeOH}}/g_{\mathrm{PVAc}}$ initially exhibit short-scale Marangoni convection cells and become convectively stable during the constant rate period.

In this work, in order to gain access to transient concentration and temperature, 1D vertical non-isothermal drying simulations have been matched to the drying curves from 3D-µPTV experiments. With this approach, it was possible to calculate transient thermal and solutal Marangoni numbers during drying, using realistic temperature- and concentration-dependent material properties, such as surface tension, viscosity, and diffusion coefficient. The results indicate that the concentration-induced instability is dominant over thermal effects, and that realistic solutal Marangoni numbers from our experiments are significantly smaller than existing critical values derived from theoretical stability analyses of stationary instability.

Finally, an empiric power-law correlation for the critical solutal Marangoni number was found (Equation (3)), with $Ma_{s,crit}\sim Pe_{int}^{2}\cdot Sc^{-1/3}$. It is in reasonable agreement with our findings, having an average relative deviation of 9.0%, and qualitatively follows trends from a theoretical stability analysis reported by Trouette et al. with regard to Péclet number and Schmidt number [72]. In future work, it should be assessed whether the correlation is valid for different material systems, drying temperatures, and higher drying rates. However, as convective Marangoni instabilities in drying polymer films may induce surface deformations, which persist in the dry film, the correlation may significantly facilitate future process design for either thin films with uniform thickness or deliberate utilizing short-scale instabilities as means for self-assembly.

## Figures and Tables

**Table 1:** Summary of all experimental conditions.

	3D Flow Field [1]	1D Concentration	Lateral Temperature Distribution	
Physical setup	3D-µPTV	IMRS	3D-µPTV	IMRS
Immersion medium	air	oil	air	oil
Substrate	glass		glass + black spray varnish coating	
Drying conditions	ambient with covering box (open top)		ambient without covering box	
Measured properties	flow field drying curve	concentration profiles drying curve	lateral film temperature	
$X_{0}/\left(g_{MeOH}/g_{PVAc}\right)$	1, 1.5, 2		1, 2	
$h_{gap}/\mu m$	50, 100, 150, 200		200	

**Table 2:** Summary of relevant properties regarding Marangoni instabilities calculated from non-isothermal simulations with *α_bottom_* = (50 W)/(m^2^K), matched to µPTV experiments, and from material properties summarized in Section 2.5. The black circles in Figure 10 indicate the source of data.

Property	Unit	Initially Stable (t≈0)	Initially Unstable (t≈0)	Stability Threshold $\left(t_{crit,Ma}\right)$
$v_{int\left(erface\right)}$	μm/s	0.23 to 0.25	0.20 to 0.29	0.19 to 0.26
$\eta \left(\bar{x},\bar{T}\right)$	Pa s	0.46 to 2.57	0.15 to 0.48	0.56 to 8.12
**Thermal Properties**				
$\Delta T=T_{surface}-T_{bottom}$	°C	−0.26 to −0.11	−0.38 to −0.10	−0.14 to −0.06
$Ma_{T}$	$10^{-3}$	2.4 to 10.0	7.0 to 206.6	0.6 to 3.3
**Solutal Properties**				
$\Delta X=X_{surface}-X_{bottom}$	$10^{-2}{\;g}_{\mathrm{MeOH}}/g_{\mathrm{PVAc}}$	−4.2 to −1.1	−3.0 to −0.8	−2.6 to −0.6
$D\left(\bar{x},\bar{T}\right)$	$10^{-10}{\;m}^{2}/s$	4.1 to 6.9	6.8 to 9.0	2.6 to 6.5
$Ma_{s}$	−	1.1 to 10.3	0.8 to 11.5	0.4 to 3.7
$Pe_{int}=v_{int}h/D$	$10^{-2}$	1.0 to 5.5	0.6 to 2.6	0.6 to 3.8
$Sc=\eta /\left(\rho \times D\right)$	$10^{6}$	0.7 to 6.6	0.2 to 0.8	0.9 to 31.6
**Comparison Thermal vs. Solutal Properties**				
$Ma_{s}/Ma_{T}$	$10^{2}$	1.9 to 10.8	0.6 to 1.9	2.4 to 49.3

## Data Availability

The data presented in this study are available in the article.

## Supplementary Materials

The following are available online at https://www.mdpi.com/article/10.3390/polym13172955/s1, Video S1: Figure 13b with animated rotation.

## Appendix A

The used material properties in this work for experiment evaluation, drying simulations, and Marangoni number calculation are summarized in the following tables. The pure component data of poly (vinyl acetate), methanol, and the glass substrate are summarized in Table A1, Table A2 and Table A3, respectively. All properties showing a strong temperature and concentration dependency are based on experimental findings in the respective references (Table A5). Properties showing a weak to no concentration or temperature dependency in the relevant range were calculated from ideal mixing rules (Table A4).

**Table A1:** Material properties of pure liquid methanol with ϑ being the temperature in °C.

Property	Unit	Value	Temperature Range/°C	Source
ρ	$\mathrm{kg}/m^{3}$	791.5	20	[1]
n	−	1.3295	20	[1]
λ	$W/\left(m\cdot K\right)$	0.20	10 to 30	[77]
$c_{p}$	$J/\left(\mathrm{kg}\cdot K\right)$	2508	20	[77]
$\Delta h_{v}$	J/kg	1,178,362	20	[77]
$p^{*}$	mbar	$10^{8.1974-\frac{1574.99}{\vartheta +238.86}}$	0 to 100	Fit to [77]

**Table A2:** Material properties of pure poly (vinyl acetate).

Property	Unit	Value	Temperature Range/°C	Source
ρ	$\mathrm{kg}/m^{3}$	1185.1	20	[82]
n	−	1.4672	20	own data (Abbemat, Dr. Kernchen)
λ	$W/\left(m\cdot K\right)$	0.159	≈20	[81]
$c_{p}$	$J/\left(\mathrm{kg}\cdot K\right)$	1449	20	[81]

**Table A3:** Thermal properties of glass substrate.

Property	Unit	Value	Temperature Range/°C	Source
ρ	$\mathrm{kg}/m^{3}$	2230	25	[83]
λ	$W/\left(m\cdot K\right)$	1.2	90	[84]
$c_{p}$	$J/\left(\mathrm{kg}\cdot K\right)$	830	20 to 100	[84]

**Table A4:** Ideal mixing rules with $x_{i}$ and $\varphi_{i}$ denoting mass fraction and volume fraction of component i, respectively.

Property	Mixing Rule Equation	Source
ρ	${\left(\underset{i}{\sum }x_{i}\cdot \rho_{i}^{-1}\right)}^{-1}$	[77]
n	$\underset{i}{\sum }\varphi_{i}\cdot n_{i}$	[4]
λ	$\underset{i}{\sum }\underset{j}{\sum }\frac{2\varphi_{i}\varphi_{j}}{\lambda_{i}^{-1}+\lambda_{j}^{-1}}$	[77]
$c_{p}$	$\underset{i}{\sum }x_{i}\cdot c_{p}$	[77]

**Table A5:** Concentration-dependent properties from poly (vinyl acetate)-methanol solutions with strong concentration dependency and with ϑ being the temperature in °C, as well as $x_{MeOH}$ and $X_{MeOH}$ being the methanol mass fraction and methanol solvent load, respectively.

Equation	Parameters	T-Range/°C	Source
$\sigma =\overset{N}{\underset{i=1}{\sum }}x_{i}\sigma_{i}+x_{i}x_{j}\left(A+B{\left[1-\left(x_{i}-x_{j}\right)\right]}^{C}\right)$	A=1089⋅ϑ+1241 B=−1089⋅ϑ−1256 $C=-8.89\cdot 10^{-6}\cdot \vartheta +4.27\cdot 10^{-4}$	10 to 40	[44]
$D_{MeOH,PVAc}=\exp\left(-\frac{A\;+\;B\cdot \;X_{MeOH}}{1+C\cdot X_{MeOH}}\right)$	A=30.39 B=111.17 C=5.57	20	[5]
$D_{MeOH,PVAc}\left(T,X\right)\;=\;D_{MeOH,PVAc}\left(T_{ref},X\right)\;\times \exp\left(-\frac{E_{A}}{\tilde{R}}\times \left(\frac{1}{T}-\frac{1}{T_{ref}}\right)\right)$ $E_{A}=\frac{d\;+\;e\times X}{1\;+\;f\;\times X}$	d=70130.82 e=140655.58 f=10.30	20 to 60	[5]
$\eta =a\times \exp\left\{\frac{b}{T}\;+\left(c_{0}+T\times c_{1}\right)\;\times \;x_{MeOH}\right\}$	$a=4.2407\cdot 10^{-8}$ b=7533.73 $c_{0}=-38.870$ $c_{1}=0.07827$	T=20 to 40 °C $x_{MeOH}=0.38$ to 1	[1]

## Appendix B

Each of the colored areas of uncertainty in Figure 10 summarize the results of nine individual curves. These curves result from the parameter variation of $\alpha_{bottom}$ (three values) in combination with calculating the Marangoni numbers with material properties using either height-averaged concentration and temperature (e.g., $\eta \left(\bar{x},\bar{T}\right)$), or local values at the bottom (e.g., $\eta \left(x_{bottom},T_{bottom}\right)$) or surface (e.g., $\eta \left(x_{surface},T_{surface}\right)$) of the film, respectively. The individual curves are given in Figure A1.
